# A predictive scoring system for postoperative delirium in the elderly patients with intertrochanteric fracture

**DOI:** 10.1186/s12893-023-02065-9

**Published:** 2023-06-08

**Authors:** Yunjiu Hu, Mingming Yang

**Affiliations:** 1 Department of Emergency Medicine, The First Affiliated Hospital of Chongqing Medical University, Chongqing, 400016 China; 2 Department of Orthopedics, The First Affiliated Hospital of Chongqing Medical University, Chongqing, 400016 China; 3 Orthopedic Laboratory of Chongqing Medical University, Chongqing, 400016 China

**Keywords:** Intertrochanteric fracture, Reduction and internal fixation, Postoperative delirium, Elderly, Prediction, Scoring system

## Abstract

**Objective:**

To establish a scoring system to predict the postoperative delirium in elderly patients with intertrochanteric fracture.

**Materials and methods:**

We retrospectively reviewed 159 elderly patients with a diagnosis of intertrochanteric fracture and underwent closed reduction and intramedullary nail fixation, and then divided them into two groups including the delirium group (23 cases) or non-delirium group (136 cases) in our hospital from January 2017 to December 2019. The following clinical characteristics were recorded and analyzed: age, gender, fracture classification, body mass index (BMI), history of diabetes mellitus, history of stroke, preoperative albumin, preoperative hemoglobin (Hb), preoperative arterial partial pressure of oxygen (PaO_2_), time between admission and surgery, lower limb thrombosis, American Society of Anesthesiologists (ASA) grade, operative time, operative blood loss, and intraoperative blood transfusion. The prevalence of these clinical characteristics in delirium group was evaluated, and the scoring system was established using logistic regression analysis. The performance of the scoring system was also prospectively validated.

**Results:**

The predictive scoring system was based on five clinical characteristics confirmed as significant predictors of postoperative delirium, namely, age > 75 years, history of stroke, preoperative Hb ≤ 100 g/L, preoperative PaO_2_ ≤ 60 mmHg, and time between admission to surgery > 3 days. Delirium group showed a significant higher score than non-delirium (6.26 vs. 2.29, *P* < 0.001), and the optimal cut-off value for the scoring system was 4 points. The sensitivity and specificity of the scoring system for predicting postoperative delirium were 82.61% and 81.62% in derivation set, respectively, and 72.71% and 75.00% in validation set.

**Conclusion:**

The predictive scoring system confirmed with achieve satisfactory sensitivity and specificity in predicting postoperative delirium in the elderly with intertrochanteric fracture. The risk of postoperative delirium in patients with the score of 5 to 11 is high, while the score of 0 to 4 is low.

## Introduction

Hip fracture is a common fracture in the elderly, accounting for about 14% of fractures in the elderly [1]. Intertrochanteric fracture is a common hip fracture in the elderly, which has a high incidence and fatality rate, causing a huge burden on the medicine, society and economy of the country [2]. With the development of surgery and anesthesia techniques, more and more elderly patients with intertrochanteric fracture choose surgical treatment [1, 2].

Postoperative delirium is an acute state of mental disorder occurring after surgery, accompanied by obvious cognitive dysfunction and impairment of attention and sleep-wake cycles, which often occurs at 24 to 72 h after surgery [3]. The elderly patients’ physiological functions are generally reduced, and they are often combined with a variety of medical diseases or malnutrition, these reasons may cause the decline of the function of tissues or organs, especially the decline of the brain function and its compensatory ability [4, 5]. At the same time, trauma response, surgical trauma and anesthesia can easily cause hemodynamic changes, leading to hypoxemia, decreased cerebral blood flow, and metabolic disorders [6]. Moreover, patients are more likely to be in a state of stress due to mental tension, braking and traction treatment during perioperative period [7]. All these above reasons increase the risk of postoperative delirium in elderly patients with intertrochanteric fracture.

It is reported that the average incidence of delirium after orthopedic surgery is 51%, and about 10–61% in elderly patients with hip fracture [8, 9]. Postoperative delirium directly affects the postoperative recovery of patients, resulting in prolonged hospital stay, increased risk of complications, mortality, and the workload of nurses and caregivers, and also waste of medical resources [8]. Therefore, to investigate the risk factors of postoperative delirium in elderly patients with intertrochanteric fracture is of great significance for reducing the risk of delirium and improving the prognosis.

At present, it is reported that postoperative delirium may be related to the following factors, such as alcoholism, underlying disease, advanced age, electrolyte disorder, type of surgery, intraoperative blood pressure fluctuation, hypoxemia, intraoperative blood loss, intraoperative fluid supplementation, gender and so on [10–12]. Besides, benzodiazepines such as diazepam and midazolam can also increase the risk of postoperative delirium [13]. But some scholars also hold different views. Scholtens et al. concluded that anesthesia method was not an independent risk factor for postoperative delirium, for there was no statistical difference in the incidence of postoperative delirium in patients undergoing intraspinal anesthesia and general anesthesia [14]. Moreover, various risk factors have different effects on the risk of delirium. Thus, a personalized assessment of the postoperative delirium risk is actually needed.

Therefore, we conducted this study to explore the risk factors for postoperative delirium in elderly patients with intertrochanteric fracture and establish a personalized predictive scoring system for postoperative delirium, so as to provide a reference for the individualized prevention and treatment of postoperative delirium.

## Materials and methods

This study was approved by the Ethics Committee of the First Affiliated Hospital of Chongqing Medical University. All of the participants provided their written informed consent to participate in this study. The work has been reported in line with the STARD criteria [15].

### Patients selection

**Fig. 1:**
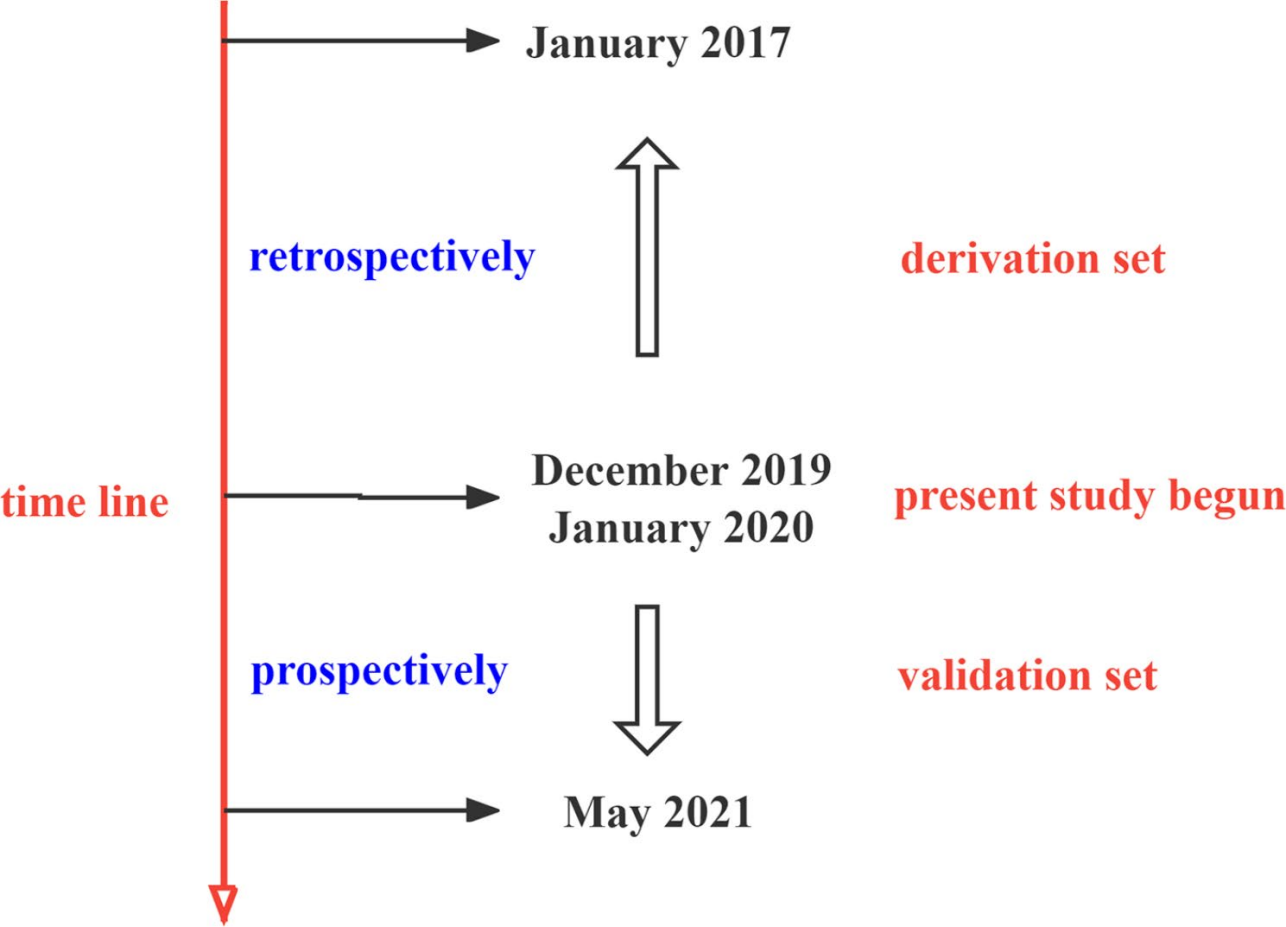
Schematic of patient inclusion in derivation and validation sets in this study

We retrospectively reviewed the medical records of hospitalized patients who were diagnosed of intertrochanteric fracture in our department from January 2017 to December 2019 to form the derivation set (Fig. 1).

Inclusion criteria: (1) Acute unilateral intertrochanteric fracture which caused by trauma (unilateral intertrochanteric fracture of the femur within one week); (2) Age ≥ 65 years old; (3) Patients who underwent closed reduction and proximal femur intramedullary nail internal fixation; (4) Postoperative follow-up time > 12 months.

Exclusion Criteria: (1) Previous history of femur surgery; (2) Multiple injuries or multiple fractures; (3) Previous history of delirium or the delirium occurred before surgery; (4) Patients with neurodegenerative diseases or mental illnesses who were disable for normal communication; (5) Patients with long-term (> 3 months) use of psychotropic medication; (6) Patients with severe underlying diseases, and cannot tolerate surgical treatment; (7) Incomplete medical record data.

### Surgical procedure

All patients were treated with PFNA internal fixation. After general anesthesia, the patient was placed on the traction bed, and the injured lower limb was fixed in traction. According to the type of fracture, the fracture was reduced by internal rotation, adduction, and longitudinal traction combined with manual pushing at the fracture site. The surgical area was disinfected and paved. A longitudinal incision was made near the apex of the greater trochanter with a length of about 4 cm. The skin, subcutaneous tissue and fascia lata were cut in turn. The gluteus medius was bluntly separated, the apex of the greater trochanter was felt, a hole was made inside the apex of the greater trochanter, and a guide needle was inserted. The C-arm X-ray fluoroscopy confirmed that the guide needle position was satisfactory. Proximal reaming was performed along the guide wire and a PFNA staple of the appropriate size was inserted. Install the sighting arm and drill the guide needle backward into the femoral neck, insert the spiral blade, and finally place a distal locking nail. C-arm X-ray fluoroscopy confirmed that the fracture reduction was satisfactory and the internal fixation position was good. The incision was rinsed, one drainage tube was placed, and the incision was sutured layer by layer.

### Data collection

Based on the results of previous studies and our experience, we included the following possible predictors for postoperative delirium, which mainly included the patient related data, preoperative data, and surgery related data.

1. Patient related data: age, gender, fracture classification, body mass index (BMI), history of diabetes mellitus, and history of stroke.
2. Preoperative data: preoperative albumin, preoperative hemoglobin (Hb), preoperative arterial partial pressure of oxygen (PaO_2_), time between admission and surgery, and lower limb thrombosis,
3. Surgery related data: American Society of Anesthesiologists (ASA) grade, operative time, operative blood loss, and intraoperative blood transfusion.
4. Follow-up clinical outcomes: during the period when patient recovered from anesthesia to discharge, the doctor referred to the confusion assessment method (CAM) to quickly identify whether the patient had postoperative delirium, and the final diagnosis was made by a psychiatrist consultation. The CAM scale mainly includes: (a) acute changes or fluctuations in consciousness; (b) attention disorder; (c) altered consciousness level; (d) mental confusion [16]. The initial diagnosis of delirium was defined as “a + b + c” or “a + b + d” on the overall assessment.

### Development of the scoring system

Firstly, all the included patients were divided into two groups, namely, delirium group and non-delirium group according to the postoperative follow-up outcomes. Secondly, univariate analysis was conducted on the patient related data, preoperative data, and surgery related data of the two groups. Based on the results of univariate analysis, the index with *P* < 0.05 was considered a possible predictor for postoperative delirium. Next, multivariate logistic regression analysis was performed for the indexes with *P* < 0.05 in univariate analysis. According to the results of multivariate logistic regression analysis, the indexes with *P* < 0.05 were considered the final predictors for postoperative delirium and, thus, determined as the items of the scoring system. Then, we established the weighted score of each item based on the relative size of Odds Ratio (OR) according to the method reported by previous research [17]. Finally, we made the appropriate cut-off points for the scoring system using ROC curves corresponding to the point on the curve nearest the upper left corner of the ROC graph.

### Validation of the scoring system

From January 2020 to May 2021, we prospectively included patients to validate the accuracy of the scoring system (Fig. 1).

The following criteria were used to determine whether a patient should be prospectively included in the validation set. Inclusion criteria: (1) Elderly patient (age ≥ 65 years) with a preoperative diagnosis of acute unilateral intertrochanteric fracture which caused by trauma. (2) Patients who had the surgical indication for closed reduction and proximal femur intramedullary nail internal fixation. Exclusion Criteria: (1) Previous history of femur surgery or delirium; (2) Multiple fractures or the delirium occurred before surgery; (3) Patients with neurodegenerative diseases, mental illnesses or long-term use of psychotropic medication.

Patients included in the study signed informed consent and then underwent closed reduction and proximal femur intramedullary nail internal fixation. Immediately after surgery, two spinal surgeons independently reviewed the clinical data, recorded the score, and predicted whether the patient would suffer from postoperative delirium according to the scoring system (predictive outcome). If there was any disagreement between the two surgeons, the consensus decision was made after a discussion with the third surgeon. During the hospital stay, the included patients were assessed whether they truly developed postoperative delirium (final follow-up outcome). The accuracy of the scoring system was evaluated by comparing the consistency between the predictive outcome and the final follow-up outcome.

### Statistical analysis

The clinical characteristics were subjected to univariate logistic regression analysis, and the significant factors were evaluated by multivariate logistic regression analysis. The items of the scoring system were determined by multivariate logistic regression, and the weighted score of each item was based on the relative size of the OR. The optimal cut-off point was made by using ROC curves. *P* < 0.05 was set of statistical significance. The SPSS version 19.0 software was used for statistical analysis.

## Results

### Derivation of the scoring system

A total of 159 patients were included in the derivation set, including 23 cases in delirium group and 136 cases in non-delirium group, and the incidence of postoperative delirium was 14.47%.

Univariate analysis showed that age > 75 years, history of stroke, preoperative albumin ≤ 35 g/L, preoperative Hb ≤ 100 g/L, preoperative PaO_2_ ≤ 60 mmHg, and time between admission to surgery > 3 days, were risk factors of postoperative delirium (Table 1).

Multivariate logistic regression analysis was carried out on the significant findings in univariate analysis and showed five clinical characteristics, namely, age > 75 years, history of stroke, preoperative Hb ≤ 100 g/L, preoperative PaO_2_ ≤ 60 mmHg, and time between admission to surgery > 3 days, were significant predictors of postoperative delirium (Table 2).

We developed a scoring system based on these five clinical characteristics that were conformed significant predictors of postoperative delirium. The variables with significant predictive value for postoperative delirium were given the weighted scores according to the relative value of the OR in multivariate logistic regression analysis: age > 75 years, history of stroke, preoperative Hb ≤ 100 g/L, preoperative PaO_2_ ≤ 60 mmHg, and time between admission to surgery > 3 days were weighted as 1 point, 3 points, 1 point, 2 points, and 1 point, respectively. The score was then calculated by determining the total number of points, ranging from 0 to 8 (Table 3).

**Fig. 2:**
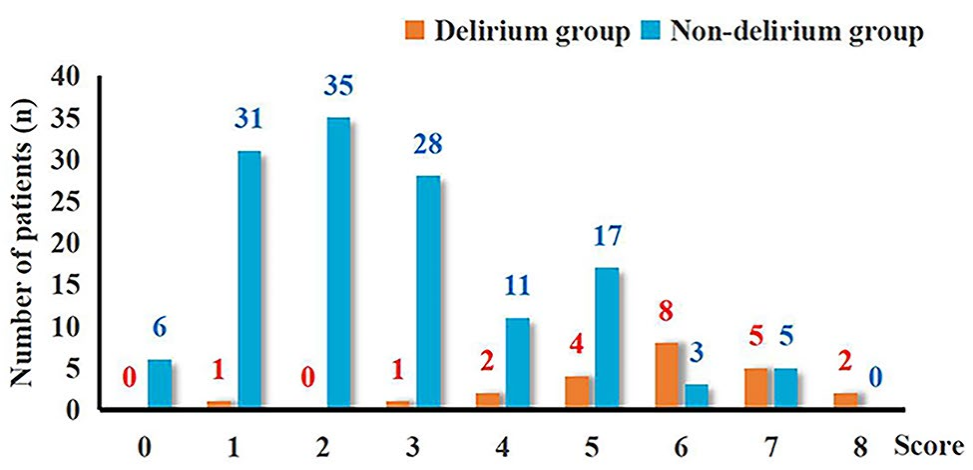
Histogram distribution of postoperative delirium and non-delirium for each score of the predictive scoring system

**Fig. 3:**
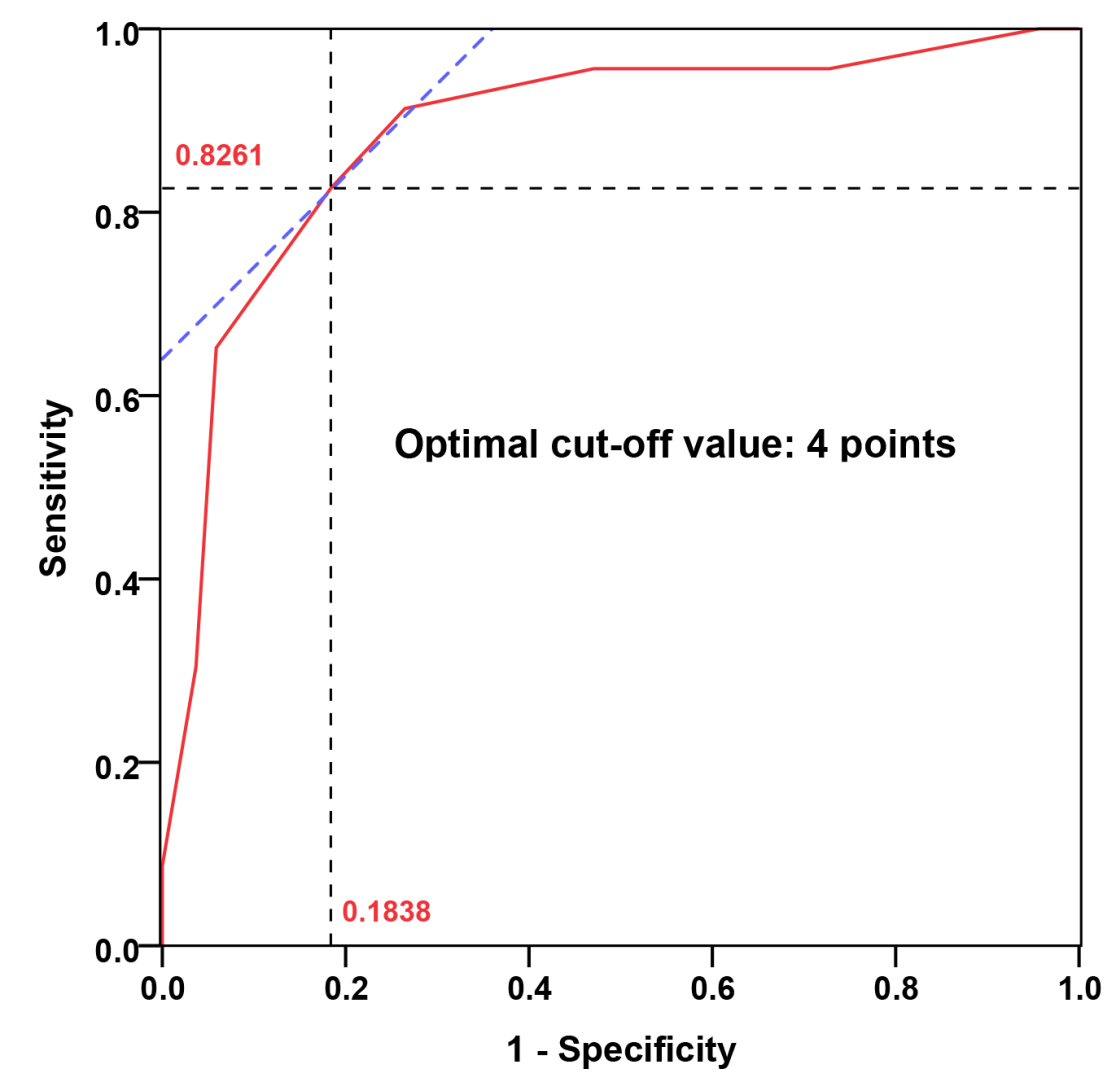
ROC curve analysis of the predictive scoring system. The optimal cut-off point based on the ROC curve analysis of scores was 4 points

A histogram distribution of the score values was shown in Fig. 2. Remarkably, delirium group showed a significant higher score than non-delirium group (6.26 ± 1.29 points vs. 2.29 ± 1.05 points, *P* < 0.001). The optimal cut-off value of the predictive scoring system was 4 points, and the area under curve (AUC) was 0.886 (95% CI: 0.807–0.965, *P <* 0.001) (Fig. 3).

**Table 1:** Univariate analysis of related variables of predicting postoperative delirium

Variables	Delirium group (N = 23)	Non-delirium group (N = 136)	P value
Age, year			0.012
≤ 75 yrs	7	81	
> 75 yrs	16	55	
Gender, n			0.653
Male	13	67	
Female	10	69	
Fracture classification (Evens), n			0.972
I	4	26	
II	4	27	
III	5	34	
IV	6	31	
V	4	18	
BMI, Kg/m^2^			0.173
≤ 20	17	79	
> 20	6	57	
Diabetes mellitus, n			0.251
Yes	6	54	
No	17	82	
History of stroke, n			0.007
Yes	8	15	
No	15	121	
Preoperative albumin, g/L			0.030
≤ 35	14	47	
> 35	9	89	
Preoperative Hb, g/L			0.018
≤ 100	16	55	
> 100	7	81	
Preoperative PaO_2_, mmHg			0.003
≤ 60	15	42	
> 60	8	94	
Time between admission to surgery, day			0.015
≤ 3	7	82	
> 3	16	54	
Lower limb thrombosis, n			0.786
Yes	6	29	
No	17	107	
ASA grade, n			0.073
I / II	16	66	
III / IV	7	70	
Operative time, min	130.5 ± 30.1	122.6 ± 23.2	0.151
Operative blood loss, ml	152.7 ± 55.4	141.1 ± 27.3	0.118
Intraoperative blood transfusion, n			0.111
Yes	14	56	
No	9	80	

BMI, body mass index; Hb, hemoglobin; PaO_2_, arterial partial pressure of oxygen; ASA, American Society of Anesthesiologists

**Table 2:** Multivariate analysis of related variables of predicting postoperative delirium

Variables	Regression coefficient (β)	Odds ratio (OR)	*P* value
Age > 75 years	0.782	2.186	0.014
History of stroke	2.013	7.486	0.006
Preoperative Hb ≤ 100 g/L	0.715	2.044	0.017
Preoperative PaO2 ≤ 60 mmHg	1.254	3.504	0.009
Time between admission to surgery > 3 days	0.805	2.237	0.013

**Table 3:** The scoring system for predicting postoperative delirium

Variable	Score
Age > 75 years	
Yes	1
No	0
History of stroke	
Yes	3
No	0
Preoperative Hb ≤ 100 g/L	
Yes	1
No	0
Preoperative PaO2 ≤ 60 mmHg	
Yes	2
No	0
Time between admission to surgery > 3 days	
Yes	1
No	0

### Validation of the scoring system

Finally, a total of 103 patients were prospectively included in the validation set, including 11 cases in delirium group and 92 cases in non-delirium group according to the follow-up clinical outcomes. Comparison of the performance of the score system on derivation set and validation set was shown in Table 4. Based on the cut-off value of 4 points, the sensitivity and specificity of the scoring system for predicting postoperative delirium were 82.61% and 81.62% in derivation set, respectively, and 72.71% and 75.00% in validation set.

**Table 4:** Comparison of performance of the scoring system on derivation set and validation set

		Derivation set			Validation set		
		Delirium (score ≥ 5)	Non-delirium (score ≤ 4)	Total	Delirium (score ≥ 5)	Non-delirium (score ≤ 4)	Total
Clinical outcomes	Delirium	19	4	23	8	3	11
	Non-delirium	25	111	136	23	69	92
	Total	44	115	159	31	72	103
Sensitivity (%)		82.61			72.73		
Specificity (%)		81.62			75.00		

## Discussion

The results of this study showed that age was an independent risk factor for postoperative delirium in elderly patients with intertrochanteric fracture. The risk of postoperative delirium was 2.186 times higher in patients over 75 years old than in patients under 75 years old, which was similar to previous studies [18, 19]. Wang et al. conducted a multiple logistic regression analysis on 582 patients undergoing joint surgery, and found that age was a major risk factor for postoperative delirium; the risk is significantly higher in patients older than 70 years than in patients younger than 70 years [19]. Through multiple regression analysis, Pinho et al. showed that the risk of postoperative delirium was higher in the elderly, suggesting that patients with delirium were more likely to have cardiac and pulmonary function complications [20]. We believe that age affects postoperative delirium mainly through the following possible reasons: (a) elderly patients had decreased cerebral cortical function, and degenerative changes occurred in the nervous system with the increase of age. These changes made the elderly more likely to suffer delirium when exposed to various stimuli [21]; (b) elderly patients have many underlying diseases, low immunity and thus showed decreased resistance to external interference factors [22]; (c) the vascular elasticity of elderly patients is low, and it is easy to suffer insufficient cerebral blood supply; the intraoperative transient hypotension may also cause serious brain tissue hypoxia, thus inducing delirium [23]. Therefore, in clinical work, attention should be paid to elderly patients, delirium risk assessment should be included in the nursing routine during hospitalization, and corresponding preventive measures should be taken actively.

History of stroke was a risk factor for postoperative delirium in elderly patients with hip fracture [24]. Guo et al. concluded that the history of stroke was one of the risk factors (OR = 5.618) for postoperative delirium via a retrospective analysis of 572 elderly patients following total hip arthroplasty (THA) for hip fracture [25]. The present study showed that stroke can increase the risk of postoperative delirium by 7.486 times in elderly patients with intertrochanteric fracture. The possible reasons were as follows: (a) after the occurrence of stroke, the increased level of cytokines in the patient’s body will up-regulate the permeability of the blood-brain barrier, and then changed the release of nerve transmission and eventually leaded to delirium [26]; (b) the remaining brain dysfunction, visual impairment and limb movement dysfunction after stroke directly affected the patient’s perception of the surrounding environment, and finally increased the risk of postoperative delirium [27]. Therefore, high attention should be paid to patients with a history of stroke, and screening of cognitive function, visual function and limb movement can help identify delirium quality and precisely.

Hypoxemia is a risk factor for postoperative delirium in elderly patients with hip fracture [28]. Hypoxia can lead to changes in the metabolic pattern of brain cells, eventually leading to brain edema, nerve cell damage, and abnormal brain function [29]. It was reported that with the increase of age, the incidence of hypoxemia also increased [30]. Shear et al. found that the blood oxygen saturation would decrease by more than 10% when a patient suffered intraoperative hypotension [31]. Therefore, while paying attention to the blood oxygen saturation of patients, it is necessary to grasp the fluctuation of their blood pressure and correct them in time. The results of this study showed that the risk of postoperative delirium in patients with hypoxemia was about 3.504 times higher than that in patients without hypoxemia. In clinical work, when a patient is transferred from the operating room to the ward after surgery, the ward doctor should consult the anesthesia record to know whether the patient suffered hypoxia during the operation. If so, the patient should be included in the high-risk group of postoperative delirium that needs focus, and oxygen saturation should be closely monitored.

It was reported that more than 48 h of preoperative preparation will significantly increased the incidence of postoperative delirium in patients with hip fracture [32]. The results of this study ndicated that preoperative preparation time longer than 3 days was an independent risk factor for postoperative delirium. Elderly patients are often complicated with a variety of internal diseases, such as reduced function of the heart, lung, liver, kidney and other organs, resulting in low stress-adaptation ability and immune function [33]. In addition, trauma and pain can also induce or aggravate internal diseases or cause water and electrolyte disorders [34]. Stabilizing medical conditions and correcting water and electrolyte disturbances often prolong the preoperative preparation time and increase the incidence of postoperative delirium. In addition of preoperative assessment and stabilization of medical diseases, poor perioperative management was also an important reason for the prolonged preoperative preparation time [35]. Therefore, it was necessary to establish an effective preoperative patient management plan and consultation system to shorten the preoperative preparation time and reduce the risk of postoperative delirium.

In conclusion, the incidence of delirium was high in elderly patients with intertrochanteric fracture, but its pathogenesis was complex and not fully understood. Advanced age was a high risk factor for postoperative delirium, but it was not controllable. Therefore, strengthening preoperative management, shortening preoperative preparation time, correcting hypoxia, anemia, low protein, water and electrolyte disorders, active nutritional support, and controlling internal medical diseases were of positive significance to reduce the risk of postoperative delirium. We believe that prevention is more important than treatment for perioperative delirium.

Our study also had limitations. First, this study was a retrospective analysis research. Second, the sample size was small. Third, other potential factors that may contribute to postoperative delirium, such as disease course and other comorbidity, were not analyzed in this study.

## Conclusion

The scoring system, which was based on five clinical characteristics, namely age > 75 years, history of stroke, preoperative Hb ≤ 100 g/L, preoperative PaO_2_ ≤ 70 mmHg, and time between admission to surgery > 3 days, seems to achieve satisfactory sensitivity and specificity in predicting postoperative delirium after intertrochanteric fracture surgery in elderly patients. The risk of postoperative delirium in patients with a score of 5 to 8 is high, while the score of 0 to 4 is low.

## Data Availability

The datasets generated and analyzed during the current study are not publicly available due to patient privacy but are available from the corresponding author on reasonable request.
